# Assessing the Relationship Between Self-Rated General Health and Colorectal Cancer Screening Uptake

**DOI:** 10.7759/cureus.74014

**Published:** 2024-11-19

**Authors:** Kelechi R Onyenemezu, Tobechukwu C Ezike, Stafford O Nwebonyi, Obinna V Ikwuka, Elochukwu V Aroh

**Affiliations:** 1 Biostatistics and Epidemiology, College of Public Health, East Tennessee State University, Johnson City, USA; 2 Internal Medicine, Nnamdi Azikiwe University Teaching Hospital, Nnewi, NGA; 3 Surgery, Chukwuemeka Odumegwu Ojukwu University Teaching Hospital, Awka, NGA

**Keywords:** colonoscopy, colorectal cancer, fit test, healthcare services, health perception, preventive health, screening uptake, self-rated general health

## Abstract

Background: Colorectal cancer screening has been shown to be effective in reducing the burden of colorectal cancer. However, the screening rate has been suboptimal, and mortality due to colorectal cancer remains high. With the presence of proactive prevention strategies, low screening rates could still be due to individual factors. Following the health-belief model, this study examined the relationship between self-rated general health perception and colorectal cancer screening.

Methods: The study utilized the 2022 Behavioral Risk Factor Surveillance System (BRFSS) Annual Survey dataset. The sample size was 177,889 and included individuals aged 45-75 years. The outcome variable was a categorical variable with binary responses of screened or not screened. The main predictor variable was an ordinal variable with five responses of poor, fair, good, very good, and excellent in the assessment of self-rated general health. Other covariates were age, sex, marital status, race, insurance status, level of education, annual income, and poor physical and mental health durations. The weighting variable of the dataset was used to adjust the sample to a weighted sample size. After obtaining descriptive statistics, simple and multivariate logistic regressions were fitted to analyze the relationship between the main predictor and the outcome variables. Statistical significance was set at a 95% confidence level.

Result: The weighted sample size was 83,303,438. Of these, 70.09% were screened and 29.91% were not screened for colorectal cancer. Excellent general health was reported by 14.80%, 34.32% very good, 32.1% good, 13.91% fair, and 4.87% had poor general health. Individuals with excellent general health perception were 25% less likely (adjusted odds ratio (AOR)=0.75; 95% CI=0.65, 0.88; p<0.0001) to be screened for colorectal cancer compared to those with poor general health perception.

Conclusion: This study identifies the individual's excellent health perception as a possible contributing factor to the deficiency towards the realization of the goal in the rate of screening uptake and the need for revised and improved awareness programs.

## Introduction

Colorectal cancer is the fourth most common cancer in the adult population of the United States, excluding skin cancer [1]. It has a mortality rate of 13 per 100,000 and accounted for 52,550 deaths (9% of all cancer deaths) in 2023, making it the second most common cause of cancer mortality [2]. Its treatment cost is the second highest among all cancers. The total medical cost for colorectal cancer in 2020 was $24.43 billion, which was 12.6% of all cancer-related treatment costs. Over 90% of that cost was accruing to medical services and hospital stay, implying an additional burden to the healthcare system [3]. Prevention of morbidity and mortality due to colorectal cancer has largely been achieved by lifestyle modification as well as by early detection and treatment through screening the average-risk population. However, screening coverage has remained suboptimal, and many cases are diagnosed late, leading to sustained high levels of morbidity and mortality. This deficit could be explained by poor motivation in the at-risk population due to poor self-rated health.

The incidence of colorectal cancer is 35.3 per 100,000, with a higher incidence in men than in women [4]. Black and American Indian people are most commonly affected. Age plays a significant role in the risk of colorectal cancer, with most cases occurring in the elderly. The screening uptake rate has been shown to increase with age [5]. Other risk factors include hereditary predisposition, sedentary lifestyle, smoking, and diet.

Over the decades, the incidence and mortality rates of colorectal cancer have progressively declined. Incidence dropped by 46% between 1985 and 2019, and mortality rate reduced by 57% between 1970 and 2020 [6]. This decline was attributed to the adoption of modifications in the risk factors such as smoking and diet. Early detection through screening in adults and treatment improvement played a major role in the success recorded in controlling the colorectal cancer burden [7]. The five-year relative survival rate from the time of diagnosis increased from 49.5% to 67.9% between 1970 and 2015 due to this [8]. A high rate of late presentation remains a concern. Sixty percent of all colorectal cancer cases between 2016 and 2020 were diagnosed at late stages, with involvement of structures outside the colon, including distant metastases [9].

The recommended minimum age for colorectal cancer screening is 45 years. Screening entails high-sensitivity stool-based tests or direct colon visualization tests. The recommended stool-based tests include guaiac fecal occult blood test (gFOBT), fecal immunochemical test (FIT), and multitarget stool DNA test (mt-sDNA). These tests are routinely repeated every one to three years after a negative result depending on the type. The direct visualization tests include colonoscopy, CT colonography, and flexible sigmoidoscopy. CT colonography and flexible sigmoidoscopy should be repeated every five years following a negative result. However, CT colonography could be repeated every 10 years when combined with a yearly negative FIT test [1]. Colonoscopy has been shown to have the highest sensitivity and specificity of all screening tests and is regarded as the gold standard for screening [10]. There is a lower rate of colorectal cancer mortality in those who undergo colonoscopy screening than in those who do not [10]. Positive stool or visualization tests, other than colonoscopy, will require a colonoscopy for further evaluation to achieve screening benefits [1]. A negative colonoscopy test requires repetition only after 10 years. Colonoscopy is a complex procedure in terms of the need for bowel preparation and skilled personnel in addition to the invasive nature of the procedure. Despite this, it is the most commonly used colorectal cancer screening modality in the United States [11]. Studies show that more than half of participants with a positive stool test result had timely follow-up with colonoscopy [12,13]. This suggests that there is a reasonable level of compliance with adequate screening processes among those who participate in colorectal cancer screening. With the Affordable Care Act in 2010, the cost of screening in the recommended average-risk population is entirely covered by all insurance providers at no cost to the patient [14].

Although overall incidence and mortality rates have steadily declined, the drop in these rates has been slower over the past two decades [6]. Between 25% and 50% of the at-risk population do not undergo screened as recommended [6]. This is shy of the 80% target of the American Cancer Society [15]. This gap has posed a challenge to the efficient control of the disease burden. Notable barriers to screening participation by the at-risk population could be implicated in this deficit. These barriers could be based on factors inherent in the individuals, sociodemographic factors, or the health system. These factors play a significant role, either independently or in combination. Health system factors include access to healthcare, complexity of recommended tests, insurance coverage, and recommendations by physicians [16,17]. Sociodemographic factors include age, gender, race, language barriers, levels of income and education, and cultural inclinations [17]. Following the health belief model of health behavior, other factors include the level of knowledge and perception of screening tests, embarrassment or discomfort due to the screening process, anxiety or fear of the unknown, disinterest, and self-rated mental or general health [17-19].

Although access and willingness to use healthcare services are remarkably higher among those who report poor self-rated health [20,21], this may not be applicable to screening services, where there is a lack of obvious ill health. People who report good self-rated health are likely want to sustain their good health by seeking out available prevention and health promotion services. They are more likely to have a better outlook and participation in screening programs compared to those who report poor self-rated health. Poor self-rated general health is an indicator of poor physical health [21,22], and it is associated with chronic diseases [22,23]. It is sensitive to a higher rate of mortality across age groups, gender, and cultures [23-26]. While those with poor self-rated health could roughly be described as being in poor physical health and needing therapeutic care, those with good self-rated health tend to be physically healthy and more proactive in preventing ill health.

A handful of European studies have identified a relationship between good self-perceived general health and colorectal cancer screening [27,28]. This relationship has raised concerns for this inquiry in the American population in light of the need to bridge the gap in the targeted rate of screening uptake in the average-risk population. Identifying this barrier will help inform targeted public health intervention to address this deficit. This study is based on the hypothesis that poor self-rated general health is associated with low levels of colorectal cancer screening participation. The objective of this study is to explore the relationship between self-rated general health and the uptake of screening.

## Materials and methods

The study sample was obtained from the Behavioral Risk Factor Surveillance System (BRFSS) survey of 2022, which is a publicly available dataset [29]. BRFSS is an annual, telephone-based, self-reported survey by the CDC that employs stratified random digit dialing. The sampling frame included non-institutionalized adults aged 18 years and older residing in the 50 U.S. states, the District of Columbia, Puerto Rico, the U.S. Virgin Islands, Guam, American Samoa, and Palau. It assessed behavioral and social determinants of health as well as the health status of individuals aged 18 years and older. This study included individuals aged between 45 and 75 years, which is the age bracket recommended for colorectal cancer screening. The analyses involved 11 variables from the dataset. The dependent variable assessed colorectal cancer screening as recommended. The primary predictor variable assessed the self-rated general health of respondents. The other covariates included age, sex, marital status, race, income level, education level, insurance, self-rated physical health, and self-rated mental health. The dataset identified individuals who met any of the recommended colorectal cancer screening requirements and coded as follows: 1=had at least one of the recommended tests within the recommended time interval, 2=did not have any of the recommended tests within the recommended time interval, and 3=have never had any of the recommended tests. These were recoded into a dichotomous outcome by merging responses 2 and 3. The new code was then defined as follows: 1=had at least one of the recommended tests within the recommended time interval and 2=did not have any of the recommended tests within the recommended time interval.

Self-rated general health was assessed as 1=Excellent, 2=Very Good, 3=Good, 4=Fair, and 5=Poor. This code was maintained for the analyses. Age was recoded as 1=age 45-49, 2=age 50-54, 3=age 55-59, 4=age 60-64, 5=age 65-69, 6=age 70-74, and 7=age 75-79, to include only the age range of interest. Sex was coded as 1=Male and 2=Female. Race was coded as 1=White, 2=Black, 3=Asian, 4=American Indian/Alaska Native, 5=Hispanic, and 6=Other races. Marital status was coded as 1=Married, 2=Divorced, 3=Widowed, 4=Separated, 5=Never married, and 6=A member of an unmarried couple. This was recoded to 1=Married and 2=Unmarried (to include response codes 2, 3, 4, 5, and 6). Health insurance was assessed using the categories: 1=Have some form of insurance and 2=Do not have some form of insurance. Income level was assessed with the codes: 1= <$15,000, 2=$15,000 to <$25,000, 3=$25,000 to <35,000, 4=$35,000 to <$50,000, 5=$50,000 to <$100,000, 6=$100,000 to <$200,000, and 7=$200,000 or more. Education level was coded as 1=Never attended school or only kindergarten, 2=Grades 1 through 8, 3=Some high school, 4=Grade 12 or GED, 5=College one year to three years (some college or technical school), 6=College four years or more (college graduate). This was recoded to 1=Little or no school (codes 1 and 2), 2=No college education (codes 3 and 4), 3=Some college education, and 4=College education or higher. Self-rated mental health and physical health were each coded as 1=Zero days when health not good, 2=One to 13 days when health not good, and 3=14+ days when health not good. Observations with missing variables were removed from the analysis. The final sample size analyzed was 177,889.

Statistical analysis

The data were analyzed using the SAS® software, Version 9.4 (2013; SAS Institute, Cary, NC, United States). Unweighted descriptive statistics of all variables was carried out to produce the frequencies and percentages of the variables. Weighted descriptive statistics was obtained using the Surveyfreq SAS procedure with the provided weighting, stratification, and clustering variables for the dataset. These variables were originally factored into the dataset and applied to appropriately justify the sample size to the population size. This application also followed the recommended instructions for analyzing the BRFSS dataset. Bivariate logistic regression analysis was performed using the surveylogistic SAS procedure to determine unadjusted odds ratios. Model selection was conducted using the backward selection method with sle=0.2 and sls=0.05. The model diagnostics also employed the Akaike Information Criterion (AIC) and receiver operating characteristic (ROC) curve C-statistic. Model selection resulted in removing the self-rated mental health variable from the subsequent multivariate analysis to control for confounding. All analyses were conducted at a 95% confidence level.

## Results

The descriptive analysis of all the variables is shown in Table 1.

**Table 1:** Weighted and unweighted frequencies of screening requirement outcome and all predictor variables.

	Frequency	Frequency percentage (%)	Weighted frequency	Weighted frequency percentage (%)
Total sample (n)	177,889	100	83,303,438	100
Screening recommendation				
Met	124,690	70.09	55,436,349	66.55
Not met	53,199	29.91	27,867,090	33.45
General health perception				
Excellent	26,327	14.80	12,255,450	14.71
Very good	61,046	34.32	27,106,866	32.54
Good	57,118	32.11	27,152,053	32.59
Fair	24,741	13.91	12,447,512	14.94
Poor	8,657	4.87	4,341,557	5.21
Age (years)				
45-49	21,984	12.36	12,602,233	15.13
50-54	25,650	14.42	15,010,955	18.02
55-59	27,516	15.47	13,910,066	16.70
60-64	32,364	18.19	16,064,360	19.28
65-69	33,893	19.05	12,614,464	15.14
70-74	30,447	17.12	11,194,963	13.44
75-79	6,035	3.39	1,906,397	2.29
Race				
White	141,076	79.31	55,359,426	66.46
Black	13,773	7.74	9,732,634	11.68
Asian	3,576	2.01	3,747,388	4.50
American Indian/Alaska Native	2,849	1.60	1,048,147	1.26
Hispanic	12,387	6.96	10,982,842	13.18
Other race	4,228	2.38	2,433,001	2.92
Sex				
Male	84,840	47.69	41,399,095	49.70
Female	93,049	52.31	41,904,343	50.30
Insurance plan				
Any insurance	171,662	96.50	79,186,465	95.06
No insurance	6,227	3.50	4,116,974	4.94
Annual income ($1,000)				
<15	10,814	6.08	5,533,126	6.64
15-24.9	16,296	9.16	7,998,320	9.60
25-34.9	18,879	10.61	8,930,396	10.72
35-49.9	21,738	12.22	9,441,274	11.33
50-99.9	56,233	31.61	24,170,761	29.02
100-199.9	40,325	22.67	19,516,635	23.43
200 or more	13,604	7.65	7,712,926	9.26
Education level				
Little or no school	2,541	1.43	2,903,292	3.49
No college	44,856	25.22	25,467,036	30.57
Some college	49,960	28.08	26,428,956	31.73
College or higher	80,532	45.27	28,504,154	34.22
Marital status				
Married	106,723	59.99	52,201,371	62.66
Not married	71,166	40.01	31,102,068	37.34
Poor physical Health				
Never	107,855	60.63	50,502,898	60.63
<2 weeks	43,490	24.45	19,866,724	23.85
≥2 weeks	26,544	14.92	12,933,816	15.53
Poor mental health				
Never	114,541	64.39	53,553,178	64.29
<2 weeks	41,721	23.45	19,071,355	22.89
≥2 weeks	21,627	12.16	10,678,905	12.82

The weighting analysis of the sample yielded a weighted sample size of 83,303,438. Of these, 50.3% were female and 49.70% were male. The sample was composed of 66.46% White participants, 11.68% Black participants, 13.18% Hispanic participants, 4.50% Asian participants, 1.26% were American Indian/Alaska Native participants, and 2.92% other races. A total of 62.66% of participants were married, while 37.34% were not married. Participants aged between 45 and 49 years were 15.15%, between 50 and 54 years were 18.01%, between 55 and 59 years were 16.70%, between 60 and 64 years were 19.28%, between 65 and 69 years were 15.14%, between 70 and 74 years were 13.44%, and between 75 and 79 years were 2.29%. Recommended screening requirements were met by 66.55%, while 29.91% did not meet the screening requirements. A total of 14.7% had excellent self-rated general health, 32.54% very good, 32.59% good, 14.94% fair, and 5.21% had poor general health. The majority of participants (95.06%) had some form of insurance, while 4.94% had no insurance. In terms of education, 3.49% had little or no school, 30.57% had no college education, 31.73% had some college education, and 34.22% had a college education or higher. The annual income assessment showed that 6.64% earned less than $15,000, 60.67% earned between $15,000 and $99,999, 23.43% earned between $100,000 and $199,999, and 9.26% earned $200,000 or more. Of the participants, 60.63% had no day of poor physical health, 23.85% experienced less than two weeks of poor physical health, and 15.53% experienced poor physical health for two weeks or more; 64.29% had no day of poor mental health, 22.90% experienced less than two weeks of poor mental health, and 12.82% experienced poor mental health for two weeks or more.

Table 2 shows the unadjusted and adjusted logistic regression analysis between the outcome and predictor variables.

**Table 2:** Bivariate and multivariate logistic regression analysis between colorectal cancer screening and the predictors. The reference category for each variable is indicated in parenthesis. For example, "(vs. White)" indicates that the White is the reference category for the race variable. The logistic regression analysis employed p<0.05 for statistical significance. OR: odds ratio.

Unadjusted (bivariate) analysis Adjusted (multivariate) analysis						
Covariates	Point estimate (OR)	Estimated confidence interval	p-value	Point estimate (OR)	Estimated confidence interval	p-value
General health perception (vs. poor health)						
Excellent	0.88	(0.79, 0.98)	0.0243	0.75	(0.65, 0.88)	<0.0001
Very good	1.09	(0.98, 1.21)	0.1042	0.87	(0.75, 1.00)	0.6093
Good	1.01	(0.91, 1.12)	0.8577	0.88	(0.76, 1.01)	0.9826
Fair	0.94	(0.84, 1.05)	0.2537	0.91	(0.80, 1.04)	0.2682
Age (years) (vs. 45-49)						
50-54	2.80	(2.58, 3.02)	<0.0001	2.98	(2.75, 3.24)	<0.0001
55-59	5.88	(5.41, 6.40)	<0.0001	6.57	(6.03, 7.16)	0.0142
60-64	7.10	(6.53, 7.72)	<0.0001	8.19	(7.50, 8.94)	<0.0001
65-69	10.13	(9.31, 11.01)	<0.0001	11.37	(10.43, 12.40)	<0.0001
70-74	11.59	(10.56, 12.73)	<0.0001	12.87	(11.70, 14.17)	<0.0001
75-79	11.70	(9.99, 13.69)	<0.0001	13.24	(11.23, 15.59)	<0.0001
Race (vs. White)						
Black	0.96	(0.90, 1.04)	0.3122	1.34	(1.24, 1.45)	<0.0001
Asian	0.55	(0.47, 0.64)	<0.0001	0.58	(0.49, 0.69)	<0.0001
American Indian/Alaska Native	0.58	(0.48, 0.70)	<0.0001	0.715	(0.57, 0.90)	0.0337
Hispanic	0.51	(0.47, 0.55)	<0.0001	0.93	(0.84, 1.02)	0.3263
Other race	0.74	(0.63, 0.86)	<0.0001	0.93	(0.77, 1.11)	0.5724
Sex (vs. male)						
Female	1.11	(1.062, 1.16)	<0.0001	1.121	(1.07, 1.18)	<0.0001
Insurance status (vs. no insurance)						
Some insurance	6.34	(5.599, 7.174)	<0.0001	3.68	(3.21, 4.23)	<0.0001
Income level ($1,000) (vs. <15)						
15-24.9	1.12	(1.00, 1.24)	0.0448	1.02	(0.90, 1.15)	<0.0001
25-34.9	1.30	(1.16, 1.44)	<0.0001	1.19	(1.05, 1.34)	0.0001
35-39.9	1.50	(1.35, 1.66)	<0.0001	1.300	(1.15, 1.47)	0.1079
50-99.9	1.77	(1.62, 1.94)	<0.0001	1.57	(1.41, 1.75)	<0.0001
100-199.9	1.66	(1.51, 1.82)	<0.0001	1.78	(1.57, 2.02)	<0.0001
200 or more	1.80	(1.61, 2.02)	<0.0001	2.14	(1.85, 2.48)	<0.0001
Education level (vs. little or no school)						
No college	1.78	(1.51, 2.09)	<0.0001	1.31	(1.06, 1.61)	0.0298
Some college	2.39	(2.04, 2.81)	<0.0001	1.611	(1.31, 1.98)	<0.0001
College or higher	2.72	(2.32, 3.19)	<0.0001	1.828	(1.48, 2.25)	<0.0001
Marital status (vs. not married)						
Married	1.39	(1.33, 1.45)	<0.0001	1.261	(1.19, 1.34)	<0.0001
Poor physical health (vs. never)						
Poor <2 weeks	1.17	(1.11, 1.24)	<0.0001	1.19	(1.12, 1.27)	0.0336
Poor ≥2 weeks	1.09	(1.02, 1.16)	0.0087	1.23	(1.12, 1.36)	0.0097

Bivariate analysis

Compared to those with self-rated poor health, those with excellent health were 12% less likely to be screened for colorectal cancer (odds ratio (OR)=0.88; 95% CI=0.79, 0.98; p=0.0243), those with very good general health were 9% more likely to be screened (OR=1.09; 95% CI=0.98, 1.21; p=0.1042), those with good health were 1% more likely to be screened (OR=1.01%; 95% CI=0.91, 1.12; p=0.8577), those with fair general health perception were 6% less likely to be screened (OR=0.94%; 95% CI=0.84, 1.05; p=0.2537). Compared to individuals aged 45-49 years, those 50-54 years old were 179% more likely to be screened (OR=2.79; 95% CI=2.58, 3.02; p<0.0001), those 55-59 years were 488% more likely to be screened (OR=5.88; 95% CI= 5.41, 6.40; p<0.0001), those 60-64 years were 610% more likely to be screened (OR=7.10; 95% CI= 6.53, 7.72; p<0.0001), those 65-69 years were 10.13 times more likely to be screened (OR=10.13; 95% CI=9.31, 11.01; p<0.0001), those 70-74 years were 11.59 times more likely to be screened (OR=11.59; 95% CI=10.56, 12.73; p<0.0001), and those 75-79 years were 11.70 times more likely to be screened (OR=11.70; 95% CI=9.99, 13.69; p<0.0001). Compared to White participants, Black participants were 4% less likely to be screened (OR=0.96; 95% CI=0.90, 1.04; p=0.3122), Asian participants were 45% less likely to be screened (OR=0.55; 95% CI=0.47, 0.64; p<0.0001), American Indian/Alaska Native participants were 49% less likely to be screened (OR=0.58; 95% CI=0.48, 0.70; p<0.0001), and other races were 23% less likely to be screened (OR=0.74; 95% CI=0.63, 0.86; p<0.0001).

Women were 11% more likely than men to be screened (OR=1.11; 95% CI=1.06, 1.16; p<0.0001). Compared to those without any insurance, those with some insurance were 534% more likely to be screened (OR=6.34; 95% CI=5.60, 7.17; p<0.0001). Compared to those with an annual income less than $15,000, those who earned between $15,000 and $24,999 were 12% more likely to be screened (OR=1.12; 95% CI=1.00, 1.24; p=0.0448), those earning $25,000-$34,999 were 30% more likely to be screened (OR=1.30; 95% CI=1.16, 1.44; p<0.0001), those earning $35,000-$49,999 were 50% more likely to be screened (OR=1.50; 95% CI=1.35, 1.66; p<0.0001), those earning $50,000-99,999 were 77% more likely to be screened (OR=1.77; 95% CI=1.62, 1.94; p<0.0001), those earning $100,000-$199,999 were 66% more likely to be screened (OR=1.66; 95% CI=1.51, 1.82; p<0.0001), and those earning $200,000 or more were 80% more likely to be screened (OR=1.80; 95% CI=1.61, 2.02; p<0.0001). Compared to those with little or no education, those with no college education were 78% more likely to be screened (OR=1.78; 95% CI=1.51, 2.09; p<0.0001), those with some college education were 139% more likely to be screened (OR=2.39 ; 95% CI=2.04, 2.81; p<0.0001), and those with a college education or higher were 172% more likely to be screened (OR=2.72; 95% CI=2.32, 3.19; p<0.0001). Compared to those who were not married, those who were married were 39% more likely to be screened (OR=1.39; 95% CI=1.33, 1.45; p<0.0001).

Compared to those who reported no day of poor physical health, those with less than two weeks of poor physical health were 17% more likely to be screened (OR=1.17; 95% CI=1.11, 1.24; p<0.0001), and those with poor physical health for two weeks or more were 9.1% more likely to be screened (OR=1.09; 95% CI=1.02, 1.16; p=0.0081). Compared to those who reported no day of poor mental health, those with less than two weeks of poor mental health were 8% less likely to be screened (OR=0.92; 95% CI=0.88, 0.94; p=0.0034), and those with poor mental health for two weeks or more were 22% less likely to be screened (OR=0.78; 95% CI=0.73, 0.84; p<0.0001).

Multivariate analysis

Compared to those with self-rated poor health, those with excellent health were 25% less likely to be screened for colorectal cancer (AOR=0.75; 95% CI=0.65, 0.88; p<0.0001), those with very good general health were 13% less likely to be screened (AOR=0.87; 95% CI=0.75, 1.00; p=0.6093), those with good health were 12% less likely to be screened (AOR= 0.88%; 95% CI=0.76,1.01; p=0.9826), those with fair general health perception were 9% less likely to be screened (AOR=0.91%; 95% CI=0.80, 1.04; p=0.2682). Compared to individuals aged 45-49 years, those aged 50-54 years were 198% more likely to be screened (AOR=2.98; 95% CI=2.75, 3.24; p<0.0001), those aged 55-59 years were 557% more likely to be screened (AOR=6.57; 95% CI=6.03, 7.16; p=0.0142), those aged 60-64 years were 719% more likely to be screened (OR=8.19; 95% CI=7.50, 8.94; p<0.0001), those aged 65-69 years were 11.37 times more likely to be screened (AOR=11.37; 95% CI=10.43, 12.40; p<0.0001), those aged 70-74 years were 12.87 times more likely to be screened (AOR=12.87; 95% CI=11.70, 14.17; p<0.0001), and those aged 75-79 years were 13.24 times more likely to be screened (AOR=13.24; 95% CI=11.23, 15.59; p<0.0001). Compared to White participants, Black participants were 33% more likely to be screened (AOR=1.33; 95% CI=1.24, 1.45; p=0.3122), Asian participants were 42% less likely to be screened (AOR=0.58; 95% CI=0.49, 0.70; p<0.0001), American Indian/Alaska Native participants were 28% less likely to be screened (AOR=0.72; 95% CI=0.57, 0.90; p=0.0337), and other races were 7% less likely to be screened (AOR=0.93; 95% CI=0.77, 1.11; p=0.5724).

Women were 12% more likely than men to be screened (AOR=1.12; 95% CI=1.07, 1.18; p<0.0001). Compared to those without any insurance, those with some insurance were 268% more likely to be screened (AOR=3.68; 95% CI=3.20, 4.23; p<0.0001). Compared to those with annual income less than $15,000, those who earned between $15,000 and $24,999 were 2% more likely to be screened (AOR=1.02; 95% CI=0.90, 1.15; p<0.0001), those earning $25,000-$34,999 were 19% more likely to be screened (AOR=1.19; 95% CI=1.05, 1.34; p=0.0001), those earning $35,000-$49,999 were 30% more likely to be screened (AOR=1.30; 95% CI=1.15, 1.47; p=0.1079), those earning $50,000-99,999 were 57% more likely to be screened (AOR= 1.57; 95% CI= 1.41, 1.75; p<0.0001), those earning $100,000-$199,999 were 78% more likely to be screened (AOR=1.78; 95% CI=1.57, 2.02; p<0.0001), and those earning $200,000 or more were 114% more likely to be screened (AOR=2.14; 95% CI=1.85, 2.48; p<0.0001). Compared to those with little or no education, those with no college education were 31% more likely to be screened (AOR=1.31; 95% CI=1.06, 1.61; p=0.0298), those with some college education were 61% more likely to be screened (AOR=1.61 ; 95% CI=1.31, 1.98; p<0.0001), and those with a college education or higher were 83% more likely to be screened (AOR=1.83; 95% CI=1.48, 2.25; p<0.0001). Compared to those who were not married, those who were married were 26% more likely to be screened (AOR=1.26; 95% CI=1.19, 1.34; p<0.0001). Compared to those who reported no day of poor physical health, those with less than two weeks of poor physical health were 17% more likely to be screened (AOR=1.19; 95% CI=1.12,1.27; p=0.0336), and those with poor physical health for two weeks or more were 23% more likely to be screened (AOR=1.23; 95% CI=1.12, 1.36; p=0.0097).

## Discussion

The study shows that the screening rate in the population is below the recommended optimal rate of 80% by the American Cancer Society [15]. This further buttresses the concern of low screening rates and the need to improve screening uptake and improve outcomes. About four-fifths of the population had good or very good general health perception, while about one-fifth reported fair or poorer health. This shows that most members of the population have a healthy perception of their general health. However, there is a statistically significant likelihood for those with poorer health to screen for colorectal cancer than those with excellent health. This relationship was further increased after controlling for confounding factors. On the other hand, those with less than excellent general health had an approximately equal likelihood of screening as those with poor health. This finding is not consistent with other studies in Europe that showed that screening is associated with better self-rated health [27,28]. These differences could be because the study in England took place before or just at the onset of the incorporation of colorectal cancer screening into the National Cancer Screening, so the attitude towards the uptake of screening in that population may not be similar to this study population, which already has routine screening as a policy. Another study only assessed the prospective intention to get screened but not the actual uptake of screening, which was assessed in this study. Furthermore, the finding seems to suggest a similarity to other studies that showed that poor self-rated general health is associated with increased access to healthcare [20,21], although that finding may not necessarily be for preventive care like screening.

There is a progressive increase in the likelihood for screening as age increases. This may be related to the fact that the elderly are at higher risk for the disease and increased awareness of screening as age increases. It is similar to other findings that showed an increasing screening rate with increasing age [5]. Higher risk could also account for the higher likelihood of screening in Black than White participants. This is in contrast to the lesser likelihood recorded among American Indian/Alaska Native participants who are also noted to be more commonly affected [5]. This finding, however, may be instrumental in highlighting a need for a more robust and targeted active intervention among this demography and to assess possible barriers to healthcare as a root cause. Although incidence is higher in men, women show more likelihood to be screened. This is consistent with other findings that women are more likely than men to obtain recommended screening services [30]. This finding is remarkable due to the known existence of more barriers for females to access healthcare than men [30]. It serves as a highlight to the possible inherent lower tendency for men to engage in preventive healthcare services.

The sampling methodology, sample size, and weighting and model diagnostic analyses are some strengths of this study. They increase the representative nature of the sample to the American population and also increase the study power. A strong limitation of the study is that it is based on a self-reported cross-sectional survey. As a result, causal inferences cannot be made from the study, and the study could be susceptible to social desirability bias. The study also did not put into consideration the varying general disposition of individuals to preventive health and health services. The possibility of general avoidance of preventive health services or health service distrust in the population is a factor worthy of consideration but not included. 

## Conclusions

The findings of this study suggest that a healthy perception of one’s general health may be a barrier to the uptake of colorectal cancer screening services. This could be informed by a lifetime history of optimal health and well-being or a negative family history of chronic diseases. This orientation may also be due to a lifestyle of exercise, diet, or other lifestyle modifications. These factors lead to reduced interface with healthcare services and may further numb the necessity of obtaining preventive services among those concerned. In addition, the presence of and participation in other health prevention initiatives and screening programs could have resulted in colorectal cancer screening being the opportunity cost arising from apparent preventive care fatigue. How self-rated general health relates to other cancer screening programs will aid in unravelling this possible concern. In any case, intensified screening sensitization programs would need to be targeted at overcoming this barrier and encourage increased screening uptake among those who have a high level of general health perception.
